# Supplementary material for: ATG4 Mediated Psm ES4326/AvrRpt2-Induced Autophagy Dependent on Salicylic Acid in Arabidopsis Thaliana
Source: Int J Mol Sci. 2020 Jul 21;21(14):5147. doi: 10.3390/ijms21145147 (PMC7404177; doi:10.3390/ijms21145147)
Supplement: Supplementary file 1 [file ijms-21-05147-s001.pdf]

International Journal of
Molecular Sciences

Supplementary data

Figure S1

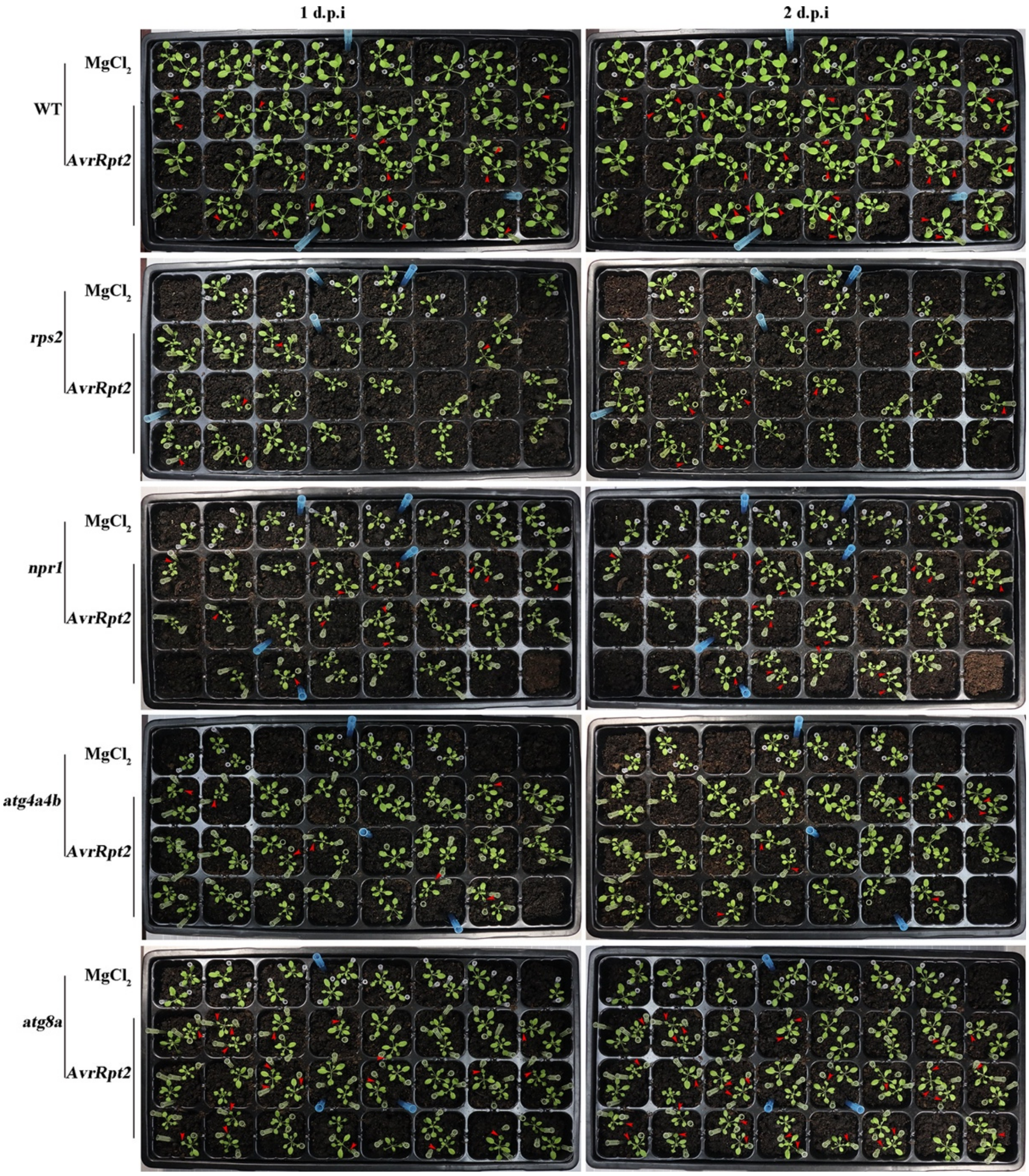

phenotypes of WT, *rps2*, *npr1*, *atg4a4b* and *atg8a* after *Psm ES4326/avrRpt2* infiltration for 1 or 2 days. The red arrow showed the leaves with HR phenotype.

Table S1 Primers for several genes.

gene name	*sequence* (5′→-3′)
AtATG1（AT3G61960）	F: ATTTGAATTCACCTGCACCA
	R: GGCAGCACTTGTTTCGTTTA
AtATG6 (AT3G61710.1)	F: TTGCAAATTCAAAGGACCAA

	R: TGGTCCAACTCTCTTGCTTG
AtATG8a (AT4G21980.1)	F: AAGCTTGGAGCTGAGAAAGC
	R: GGAACCCATCCTCATCTTTG
ICS1 (AT1G74710.2)	F: GGCAGGGAGACTTACG
	R: AGGTCCCGCATACATT
NPR1 (AT1G64280.1)	F: CAATTCATCGGAACCTGTTG
	R: GAGGAGTCGGTGTTATCGGT
PR1 (AT2G14610)	F:CTCAAGATAGCCCACAAGAT
	R:GCGTAGTTGTAGTTAGCCTTCT
EDS1 (AT3G48090)	F:CCAATTGGATCCCAGAAAGT
	R:AACAGCTTGGTTTGCAACAG
ubiquitin 5	F:GCCGAAGAAGATCAAGCACAAGCA
	R:TCGATGGATCTGGAAAGGTTCAGCGT
AtACTIN2	F: GGTAACATTGTGCTCAGTGGTGG
	R: AACGACCTTAATCTTCATGCTGC
